# miR-263b Controls Circadian Behavior and the Structural Plasticity of Pacemaker Neurons by Regulating the LIM-Only Protein Beadex

**DOI:** 10.3390/cells8080923

**Published:** 2019-08-18

**Authors:** Xiaoge Nian, Wenfeng Chen, Weiwei Bai, Zhangwu Zhao, Yong Zhang

**Affiliations:** 1 Department of Entomology and MOA Key Lab of Pest Monitoring and Green Management, College of Plant Protection, China Agricultural University, Beijing 100193, China; 2 Department of Biology, University of Nevada Reno, Reno, NV 89557, USA; 3 Institute of Life Sciences, Fuzhou University, Fuzhou 350108, China

**Keywords:** *Drosophila*, microRNA, circadian rhythms, structural plasticity, Beadex

## Abstract

Circadian clocks drive rhythmic physiology and behavior to allow adaption to daily environmental changes. In *Drosophila*, the small ventral lateral neurons (sLNvs) are primary pacemakers that control circadian rhythms. Circadian changes are observed in the dorsal axonal projections of the sLNvs, but their physiological importance and the underlying mechanism are unclear. Here, we identified *miR-263b* as an important regulator of circadian rhythms and structural plasticity of sLNvs in *Drosophila*. Depletion of *miR-263b* (*miR-263b^KO^*) in flies dramatically impaired locomotor rhythms under constant darkness. Indeed, *miR-263b* is required for the structural plasticity of sLNvs. *miR-263b* regulates circadian rhythms through inhibition of expression of the LIM-only protein *Beadex (Bx).* Consistently, overexpression of *Bx* or loss-of-function mutation (*Bx^hdpR26^*) phenocopied *miR-263b^KO^* and *miR-263b* overexpression in behavior and molecular characteristics. In addition, mutating the *miR-263b* binding sites in the *Bx* 3′ UTR using CRISPR/Cas9 recapitulated the circadian phenotypes of *miR-263b^KO^* flies. Together, these results establish *miR-263b* as an important regulator of circadian locomotor behavior and structural plasticity.

## 1. Introduction

Circadian clocks are intracellular pacemakers that generate approximately 24-h rhythms of behavior and physiology in most organisms. In animals, the core circadian oscillator consists of a conserved autoregulatory transcriptional–translational negative feedback loop [1,2,3]. The fruit fly, *Drosophila melanogaster*, serves as a great model in dissecting the molecular and neuronal mechanisms of circadian clocks [2,3,4]. In *Drosophila*, transcription factors CLOCK (CLK) and CYCLE (CYC) activate the rhythmic transcription of clock-controlled genes. Among these genes, PERIOD (PER) is a key repressor in the core circadian negative feedback loop that inhibits CLK/CYC activity and represses per transcription. Post-translational modifications such as phosphorylation, glycosylation, and ubiquitination also play important roles in setting the pace of circadian clock [2,5,6,7,8,9].

Circadian locomotor rhythms of flies are generated by a neuronal network consisting of about 150 brain circadian neurons [4,10,11,12]. Based on their relative location and function, these circadian neurons are divided into several clusters: three groups of ventral lateral neurons (LNvs), three groups of dorsal neurons (DN1s, DN2s, and DN3s), dorsal lateral neurons (LNds), and lateral posterior neurons (LPNs). The large LNvs (lLNvs) and four of the small LNvs (sLNvs) express the neuropeptide pigment dispersing factor (PDF), whereas the fifth sLNv is PDF-negative [12]. Under regular 12-h:12-h light–dark (LD) conditions at 25 °C, flies exhibit bimodal locomotor activity rhythms, peaking around dawn and dusk. PDF-positive sLNvs are responsible for promoting the morning peak before daylight, and the fifth sLNv and the LNds are mainly responsible for generating the evening activity [13,14]. DN1s can integrate light and temperature inputs to generate either morning or evening activity; DN1s also promote sleep [15,16]. A recent study indicated that the activity of DN1s is acutely modulated by temperature; thus, DN1s sense temperature to regulate sleep [17]. On the other hand, DN2s play important roles in the temperature preference of circadian rhythm [18].

The organization of the circadian neuronal network is similar in flies and mammals. The suprachiasmatic nucleus (SCN) of the hypothalamus is the mammalian primary circadian pacemaker, which synchronizes other neurons and generates circadian locomotor rhythms [19,20]. As important pacemaker neurons in flies, the PDF-positive sLNvs communicate with other circadian neurons, thus enabling flies to maintain robust circadian locomotor rhythms in constant conditions [21]. Elimination of sLNvs or mutation of *Pdf* or its receptor causes flies to be largely arrhythmic, and shortens the circadian period in the rhythmic flies [21,22]. The PDF-positive sLNvs also send axonal projections toward the dorsal protocerebrum of the fly brain, where DN1s and DN2s are located [23,24]. PDF immunoreactivity in the dorsal axonal terminal of sLNvs has a clearly circadian pattern with high intensity during the daytime and low intensity at nighttime [25]. The sLNvs dorsal projections also show arborization rhythms with higher complexity of axon terminals found in the early day and lower complexity at night [26]. This structural plasticity of the sLNv dorsal projections is controlled by both circadian- and activity-dependent mechanisms [27,28]. The molecular mechanisms underlying this plasticity of sLNv are poorly understood, however. The transcription factor Mef2 was found to control circadian plasticity of sLNvs through regulation of the cell adhesion molecule Fas2 [28]. In addition, two matrix metalloproteinases (MMP1, and MMP2) were also shown to be required for the structural remodeling control of sLNv projections [29]. Rhythmic Rho1 activity was identified to regulate the sLNvs neuronal plasticity [30]. Recently, the circadian protein VRILLE was found to control sLNv arborization rhythms through unknown mechanisms [31].

MicroRNAs (miRNAs) are small non-coding RNAs that regulate gene expression post-transcriptionally [32]. miRNAs repress expression of their target genes through messenger RNA (mRNA) degradation and/or translation inhibition. Emerging evidence shows that miRNAs are involved in many important physiological and pathological conditions, such as cardiac and pulmonary diseases and even cancer [33,34,35]. Recent studies uncovered critical roles of miRNAs in the regulation of different aspects of circadian rhythms [36,37]. In mouse, *miR-132/212* modulates circadian entrainment to day length, thus influencing seasonal adaptation, while *miR-219* and *miR-24* affect circadian period length [38,39]. In *Drosophila*, overexpression of the miRNA *bantam* lengthens circadian locomotor period by repressing *Clk*, while pacemaker genes *timeless* and *clockwork orange* are targets of *miR-276a* and *let-7*, respectively [40,41,42]. miRNAs also play important roles in circadian output pathways. We showed previously that the disruption of general miRNA functions resulting from depletion of GW182, a key protein for miRNA-mediated gene silencing, affects circadian rhythms by interfering with PDF-receptor signaling [43]. In addition, the *miR-279* and *miR959-964* cluster miRNAs modulate circadian locomotor behavior output and circadian timing of feeding and immune responses [44,45]. In addition to us, others also demonstrated that *miR-124* controls the phase of circadian locomotor activity [46,47].

Here, we show that a conserved miRNA, *miR-263b*, is an important regulator of circadian rhythms in *Drosophila*. *miR-263b* regulates circadian locomotor behavior by affecting the structural plasticity of sLNvs through inhibition of *Beadex* (*Bx*) expression.

## 2. Results

### 2.1. *miR-263b* Regulates Circadian Behavior

**Figure 1:**
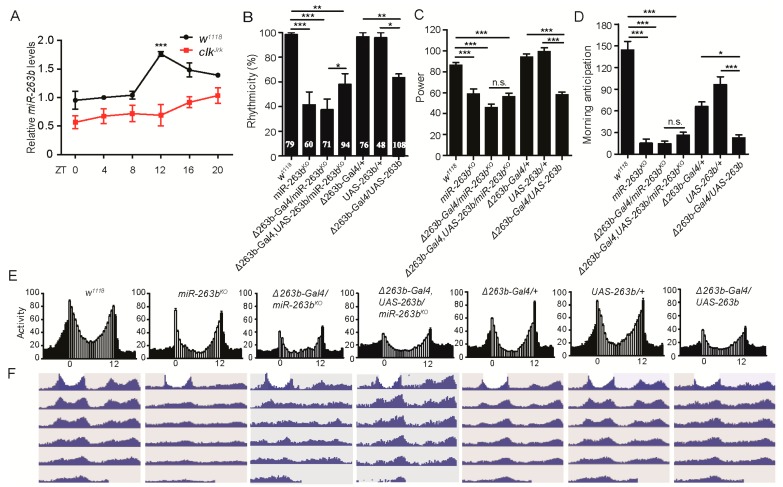
*miR-263b* is rhythmically expressed and regulates circadian behavior. (**A**) Quantitative PCR analysis of *miR-263b* from adult brains of wild-type and *Clk^Jrk^* mutant flies at the indicated time points. The relative expression levels were normalized to *2s ribosomal RNA* (rRNA) and were further normalized to *w^1118^* at ZT0. Each time point was compared to ZT0. Data represent means ± standard error of the mean (SEM); *** *p* < 0.001 determined by Student’s *t*-test. (**B**–**D**) Comparison of (**B**) rhythmicity, (**C**) power, and (**D**) morning anticipation index in indicated fly strains. Data represent means ± SEM (*n* = 45–79); * *p* < 0.05, ** *p* <0.01, *** *p*< 0.001 determined by Student’s *t*-test. (**E**) Locomotor activity of indicated strains measured during three days of light–dark (LD). The white and black bars indicate day and night, respectively. (**F**) Actograms showing the average activities on the last day in LD and five days in constant darkness (DD). Light represents the day, and gray represents the darkness.

To identify miRNAs that regulate circadian rhythms, we performed a screen for all of the available miRNA mutants in the *Drosophila* stock center [48]. *miR-263b* is among the miRNAs that exhibit strong circadian phenotypes. It was observed that *miR-263b* has circadian oscillations by microarray [49]; thus, we decided to focus on *miR-263b*. We firstly used miRNA quantitative real-time PCR to confirm the oscillation of *miR-263b*. We found that the expression of *miR-263b* is rhythmic in fly heads, consistent with previous data that were published (Figure 1A [49]). Importantly, the oscillation of *miR-263b* was abolished in the *Clk^Jrk^* mutant, confirming that the expression of *miR-263b* is under circadian control (Figure 1A).

To examine the circadian role of *miR-263b*, we analyzed the *miR-263b* knockout (*miR-263b^KO^*) flies [50]. In constant darkness (DD) condition, flies lacking *miR-263b* displayed a severely disrupted circadian locomotor rhythm (Table 1). In contrast to the wild-type flies, more than 60% of the *miR-263b^KO^* flies became arrhythmic (Figure 1B). Moreover, the amplitude of behavioral rhythms was clearly reduced even in rhythmic *miR-263b^KO^* flies (Figure 1C). Because of the high percentage of arrhythmia of the *miR-263b^KO^* flies in DD, we examined the locomotor activity under light–dark cycles (LD). Under LD, wild-type flies gradually increase their activity before light on, an anticipatory behavior controlled by the clock. In *miR-263b^KO^* flies, however, the morning anticipation was abolished (Figure 1D,E).

To confirm that the disruption of circadian rhythms is caused by depletion of *miR-263b*, we utilized a *Gal4* knock-in replacement (*Δ263b-Gal4* [50]). *Δ263b-Gal4*/*miR-263b^KO^* flies showed similar circadian behavior phenotypes as *miR-263b^KO^* flies (Figure 1B–D and Table 1). Next, we aimed to rescue the circadian rhythm defects of *miR-263b^KO^* by expressing *miR-263b* using *Δ263b-Gal4*. Overexpression of *miR-263b* by *Δ263b-Gal4* resulted in a ~9-fold increase of *miR-263b* abundance (Figure S1, Supplementary Materials). We observed a partial but significant rescue of rhythmicity of the circadian locomotor behavior (Figure 1B). However, overexpression of *miR-263b* in wild-type flies also disrupted circadian locomotor activity rhythms in DD, and reduced morning anticipation (Figure 1B–E). Notably, the free-running period was not significantly altered in the flies in which *miR-263b* levels were abnormal (Figure 1F and Table 1).

To study the temporal requirement of *miR-263b*, we used a conditional temperature-sensitive *tubulin-Gal80^ts^* repressive transgene coupled with the *Δ263b-Gal4* driver to overexpress *miR-263b* at different developmental stages. Adulthood specific expression of *miR-263b* caused ~50% of the flies to be arrhythmic (Figure S2, Supplementary Materials). By contrast, flies with overexpression of *miR-263b* during development exhibited slightly reduced rhythmicity, which was not significantly different from the *UAS-263b* control (Figure S2, Supplementary Materials). Together, these data indicate that *miR-263b* is predominantly required at adulthood for circadian locomotor rhythms.

### 2.2. *miR-263b* Is Required for the Circadian Structural Plasticity of sLNvs Axonal Projections

To examine whether the impaired circadian locomotor rhythmicity in *miR-263b^KO^* flies is due to a defect in the molecular pacemaker or in the circadian output pathway, we examined the oscillation of the key pacemaker protein PER in DD in three important groups of circadian neurons: sLNvs, LNds, and DN1s. We found no obvious changes in PER abundances or oscillation in these neurons known to control locomotor behavior (Figure S3, Supplementary Materials), suggesting that *miR-263b* may regulate circadian behavior by modulating the output pathway. 

**Figure 2:**
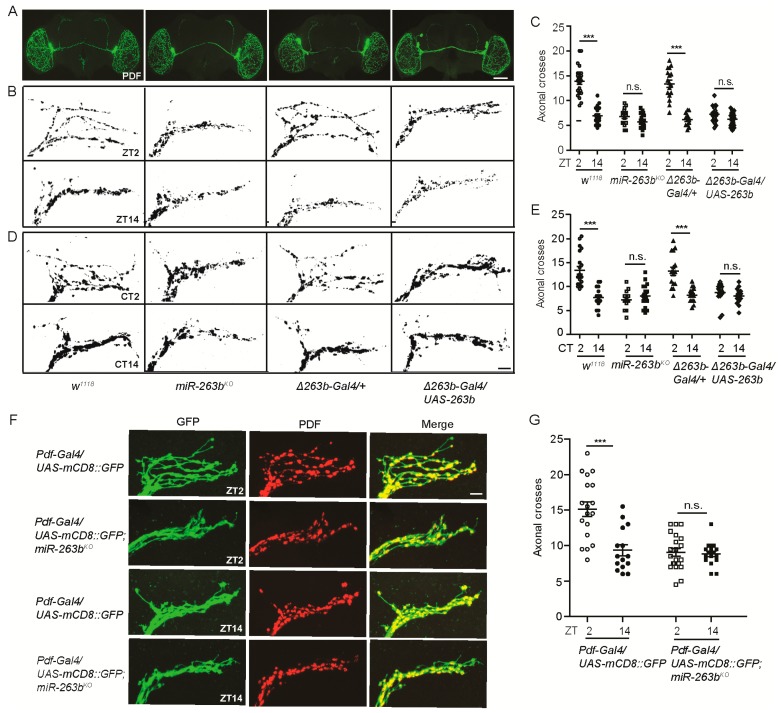
*miR-263b* modulates the structural plasticity of small ventral lateral neuron (sLNv) axonal projections. (**A**) Representative confocal images of whole brains from the indicated genotypes stained for pigment dispersing factor (PDF) at Zeitgeber time 2 (ZT2); scale bar, 75 µm. (**B**) Representative images of sLNv dorsal projections from the indicated genotypes stained with anti-PDF at ZT2 and ZT14. Flies were entrained for at least three days under LD conditions prior to the assay. Note the “open”, defasciculated axonal conformation at ZT2 and fasciculated axons at ZT14 in control w1118 flies; scale bar, 10 µm. (**C**) Quantification of axonal morphology (fasciculation) of sLNv dorsal termini in LD conditions by Sholl’s analysis (see also Methods). Data represent means ± SEM (*n* = 15–22); n.s. not significant, *** *p* < 0.001 determined by Student’s *t*-test. (**D**) Representative images of sLNv dorsal projections from the indicated genotypes stained with anti-PDF at circadian time 2 (CT2) or CT14 during the second day of DD; scale bar, 10 µm. (**E**) Quantification analysis of axonal morphology of sLNv dorsal termini in DD conditions. Data represent means ± SEM (*n* = 15–21); n.s. not significant, *** *p* < 0.001 determined by Student’s *t*-test. (**F**) Structural plasticity of sLNv dorsal projections is regulated by *miR-263b*. Axonal projection of sLNv labeled by a membrane-tethered GFP. Representative confocal images of fly brains stained for GFP (green) and PDF (red) dissected at ZT2 and ZT14; scale bar, 10 µm. (**G**) Quantification analysis of axonal morphology of sLNv dorsal termini. Data represent means ± SEM (*n* = 15–21); n.s. not significant, *** *p* < 0.001 determined by Student’s *t*-test.

Circadian structural remodeling of sLNvs dorsal projections was observed [26]. To test the hypothesis that *miR-263b* controls the circadian plasticity of PDF-positive projections, we examined the termini of sLNv dorsal projections in *miR-263^KO^* flies with a PDF-specific antibody at early day (Zeitgeber time 2 (ZT2), ZT0 is light on and ZT12 is light off) and early night (ZT14). Consistent with previous reports, we found that the wild-type flies had more branches of axon terminals at ZT2 than ZT14 (Figure 2A–C). The axonal crosses were quantified with Sholl’s analysis to assay the interactions between axon branches and concentric circles (see Methods [28]). Remarkably, this structural plasticity was abolished in the *miR-263b^KO^* flies due to a significant decrease of axonal crosses at ZT2 compared to control *w^1118^* flies (Figure 2A–C). Interestingly, overexpression of *miR-263b* caused similar defects in PDF axonal projections (Figure 2A–C). These data indicate that the proper level of *miR-263b* is required for the circadian structural plasticity of sLNv dorsal projections. It was reported that the structural plasticity of sLNvs is both under circadian regulation and activity-dependent [26,28,51]. To further confirm that *miR-263b* affects the circadian structural plasticity of sLNvs, we dissected fly brains in DD. In wild-type flies, we observed more axonal branches at circadian time 2 (CT2) in the subjective morning and fewer branches in the subjective night (CT14) (Figure 2D,E). Similar to the results in LD, the rhythmic change in structural plasticity was abolished in the *miR-263b^KO^* flies and in flies with overexpression of *miR-263b* in DD (Figure 2D,E). To test whether sLNv dorsal projections were present in the absence of *miR-263b*, a membrane-tethered GFP (mCD8::GFP) was used to mark the PDF projections under the control of a *Pdf*-specific promoter. As observed by PDF staining, this marker revealed that the dorsal axonal branches of sLNvs were dramatically reduced (Figure 2F,G). Furthermore, no significant changes of PDF levels were detected in the sLNvs soma, indicating that the decrease of axonal branches was not due to the decrease of PDF staining (Figure S4, Supplementary Materials). To test whether *miR-263b* can drive changes of sLNv axonal projections acutely, we used the temperature-sensitive *Gal80^ts^* to temporally induce *miR-263b* overexpression at ZT18, and dissected the relative fly brains at ZT2 next day (6 h after induction). Compared to the *Gal4* control, overexpression of *miR-263b* for 6 h significantly reduced the structural plasticity of sLNvs (Figure S5, Supplementary Materials), which indicates the acute function of *miR-263b*.

**Figure 3:**
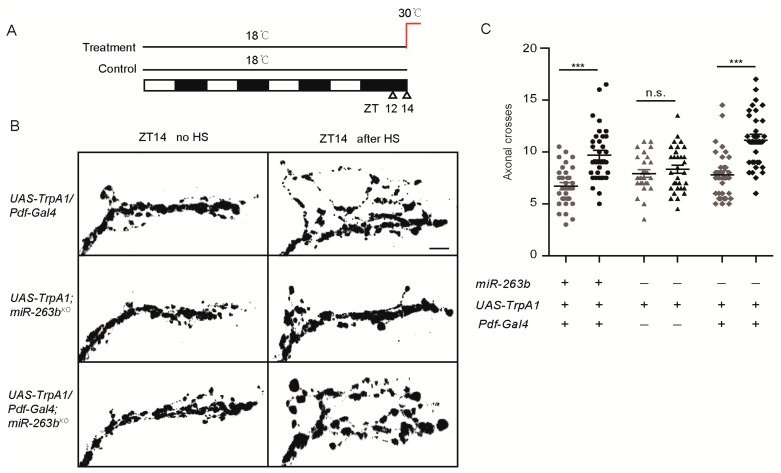
Activity-dependent remodeling of sLNv axonal fasciculation is intact in *miR-263b^KO^* flies. (**A**) Diagram of activity induction by heat. Flies were raised at 18 °C and entrained in LD cycles at 18 °C for at least three days before shifting to 30 °C at ZT12. Flies were dissected 2 h later (ZT14) for anti-PDF staining. (**B**) Representative confocal images of sLNv projections at ZT14 with or without heat shock (HS). Induction of *TrpA1* in PDF cells by 2-h temperature elevation to 30 °C leads to open conformation of sLNv dorsal projections at ZT14 in all strains; scale bar, 10 µm. (**C**) Quantification of *TrpA1*-induced changes in axonal fasciculation. “+” or “−” represents that the relative genes are present or absent in the flies. Data represent means ± SEM (*n* = 26–34); n.s. not significant, *** *p* < 0.001 determined by Student’s *t*-test.

sLNv axonal complexity is modified in response to electrical activity, and adult-specific activation of sLNvs results in increased complexity [27]. Thus, we examined whether this activity-dependent structural plasticity requires *miR-263b.* We took advantage of the temperature-gated *TrpA1* channel to increase the neuronal activity of sLNvs [52]. Increasing the environment temperature to 30 °C for 2 h beginning at ZT12 specifically activated sLNvs and caused defasciculation of sLNv dorsal termini at ZT14 in flies expressing *TrpA1* (Figure 3A,B). Activation of sLNvs by temperature treatment also increased the dorsal projection complexity (Figure 3B,C). Effects were dependent on *TrpA1* expression and were similar in all genotypes, including *miR-263b^KO^*. Together, these results indicate that *miR-263b* is required for the circadian structural plasticity but dispensable for the activity-dependent rhythms in sLNv dorsal projections.

### 2.3. *miR-263b* Is Required in the PDF-Positive sLNvs for Circadian Behavior

**Figure 4:**
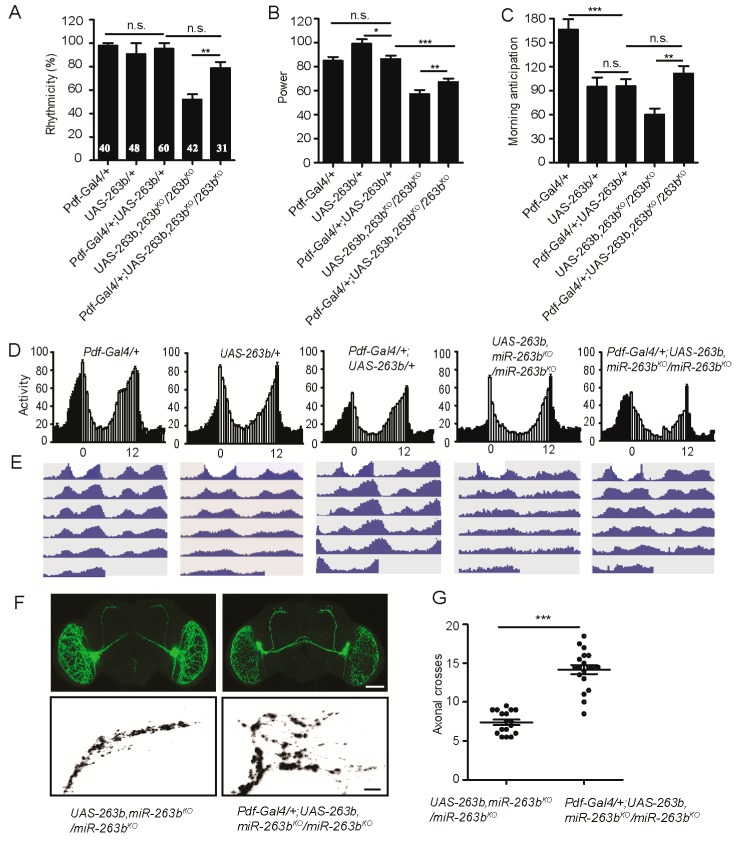
Expression of *miR-263b* in PDF-positive neurons rescued *miR-263b* defect on circadian rhythmicity and structural plasticity. (**A**–**C**) Comparison of (**A**) rhythmicity, (**B**) power, and (**C**) morning anticipation index in indicated fly strains. Data represent means ± SEM (*n* = 45–79); * *p* <0.05, ** *p* <0.01, *** *p* <0.001 determined by Student’s *t*-test. (**D**) Locomotor activity of indicated strains measured during three days of LD. The white and black bars indicate day and night, respectively. (**E**) Actograms showing the average activities on the last day in LD and five days in DD. Light represents the day, and gray represents darkness. (**F**) Representative confocal images of whole brains (scale bar, 75 µm.) and sLNv dorsal projections (scale bar, 10 µm.) from the indicated genotypes stained for PDF at ZT2. (**G**) Quantification of axonal morphology (fasciculation) of sLNv dorsal termini in LD conditions by Sholl’s analysis (see also Methods). Data represent means ± SEM (*n* = 17–19); *** *p* < 0.001 determined by Student’s *t*-test.

Since *miR-263b* regulates the structural plasticity of sLNv neurons, we wondered whether *miR-263b* is required in the sLNvs for the circadian behavior. *Pdf-Gal4* was used to express *miR-263b* in the *miR-263b* mutants. As we expected, restoration of *miR-263b* in sLNv neurons by *Pdf-Gal4* significantly increased the rhythmicity of *miR-263b* mutants (Figure 4A). Indeed, the rhythmicity in the rescue was insignificant from the controls (Figure 4A). The power of rhythms and the morning anticipation in *miR-263b* mutants were also restored (Figure 4B–E). Furthermore, not only the circadian locomotor rhythm, but also the expression of *miR-263b* in the PDF-positive cells increased the axonal crossings at ZT2, thereby rescuing the defects of structural plasticity in *miR-263b* mutants (Figure 4F,G). Taken together, our results indicate that *miR-263b* is necessary in the PDF-positive sLNvs for circadian behavior and structural plasticity.

### 2.4. *Bx* Regulates Circadian Rhythms, and Its Expression Is Suppressed by *miR-263b*

Next, we used an in silico prediction algorithm to identify putative *miR-263b* targets by using the annotated *Drosophila* genome (http://www.targetscan.org/fly_12/). Among the approximate 400 potential targets predicted, *Beadex* (*Bx*), which encodes a LIM-only protein, was one of the top 16 candidates, based on the potential binding affinity. In addition, target prediction indicated that the *Bx* 3′ UTR has two putative *miR-263b* binding sites, which are highly conserved across *Drosophila* species (Figure S6, Supplementary Materials). Since disrupted circadian rhythms were previously reported in flies with *Bx* mutations [53], we decided to focus on *Bx*.

**Figure 5:**
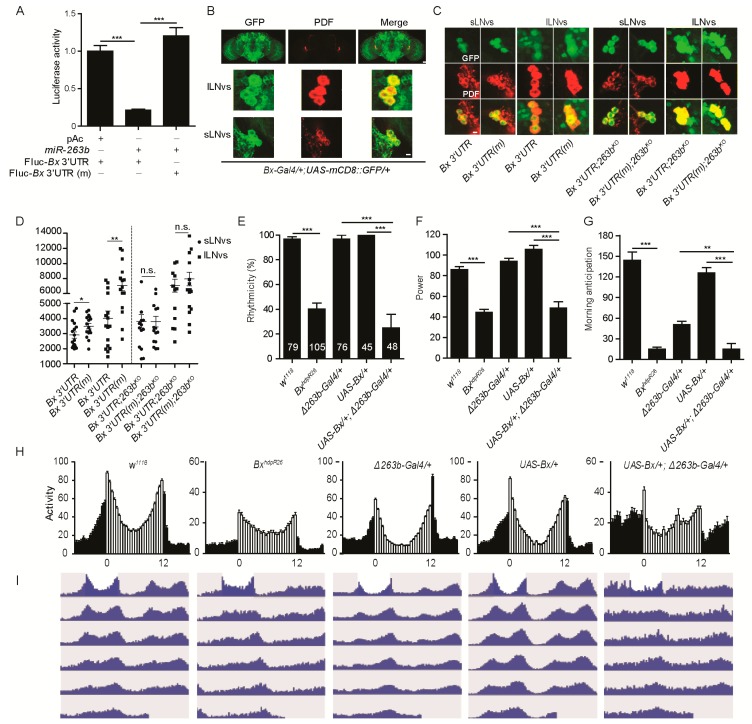
*Bx* regulates circadian rhythms, and its expression is suppressed by *miR-263b.* (**A**) *miR-263b* inhibited the expression of *Bx* in S2 cells. pAC or pAC-*miR-263b* were co-transfected with pAc-fluc-*Bx* 3′UTR or pAc-fluc-*Bx* 3′ UTR (mut) and with pCopia-Renilla luciferase into S2 cells. After two days, luciferase activity was quantified. For each condition, a normalized firefly/*Renilla* luciferase value is plotted with SEM. (**B**) *Bx* is expressed in the PDF-positive sLNvs and large LNVs (lLNvs). Representative confocal images of fly brains (scale bar, 75 µm), lLNvs, and sLNvs (scale bar, 10 µm) stained for GFP (green) and PDF (red) dissected at ZT13. (**C**) Representative confocal images of *Bx* 3′ UTR (*Bx-GAL4/+; UAS-EGFP-Bx 3′UTR/+*) and *Bx* 3′ UTR (mut) (*Bx-GAL4/+; UAS-EGFP-Bx 3′ UTR(mut)/+*) in wild-type or *miR-263b* mutant background. The brains were stained with anti-GFP antibody (green) and anti-PDF antibody (red) at ZT13; scale bar, 10 µm. (**D**) Quantification of GFP signals in sLNvs and lLNvs from *Bx* 3′ UTR and *Bx* 3′ UTR (mut) flies. Data represent means ± SEM (*n* = 16–17); * *p* < 0.05, ** *p* < 0.01 determined by Student’s *t*-test. (**E**–**G**) Comparison of (**E**) rhythmicity, (**F**) power, and (**G**) morning anticipation in *indicated* lines. The numbers of tested flies are shown in each column. Each experiment was conducted three times. Data represent means ± SEM (*n* = 45–105); ** *p* < 0.01, *** *p* < 0.001 determined by Student’s *t*-test. (**H**) Locomotor activity of adult male flies of the indicated genotypes measured during three days of LD cycle. The white and black bars indicate day and night, respectively. (**I**) Actograms showing the average activities on the last day in LD and the five days in DD. Light represents the day, and gray represents darkness.

To test whether *miR-263b* directly binds to the *Bx* 3′ UTR and inhibits its expression, the *Bx* 3′ UTR was fused downstream of a luciferase reporter and transfected into S2 cells. Luciferase activity was significantly suppressed when *miR-263b* was co-transfected with the reporter. In contrast, the luciferase activity was observed in the presence of *miR-263b* when the putative *miR-263b* binding sites within the *Bx* 3′ UTR were mutated (Figure 5A). Next, we determined whether *Bx* is expressed in the sLNvs by labeling *Bx*-expressing neurons (*UAS-mCD8::GFP/+; Bx-Gal4/+*) with GFP and immunolabeling with anti-PDF antibody. Results showed that *Bx* was broadly expressed in the fly brain. Interestingly, strong GFP signals in PDF-positive sLNv and lLNv neurons were clearly observed (Figure 5B). Encouraged by these data, we further tested whether *Bx* abundance is suppressed by *miR-263b* in fly brain. We tested whether *miR-263b* regulates Bx levels by expressing an enhanced GFP (EGFP) reporter fused to the wild-type *Bx* 3′ UTR or 3′ UTR with *miR-263b* binding sites mutated (same construct as Figure 5A). We used a site-specific integration approach to insert the *Bx-*3′ UTR wild-type and mutant constructs in the same chromosomal location and made transgenic fly lines. We expressed these two reporters using the *Bx-Gal4* line that we used for staining. Strikingly, we found that the expression of EGFP under the control of the wild-type *Bx* 3′ UTR was significantly lower than the mutant 3′ UTR in both sLNvs and lLNvs. Furthermore, the differences in EGFP expression between wild-type and mutant *Bx* 3′ UTR were abolished under *miR-263b^KO^* background (Figure 5C,D). Thus, our S2 cell and imaging results suggest that *Bx* is a direct target of *miR-263b* and is negatively regulated by *miR-263b*.

miRNAs are normally negative regulators of gene expression; since we observed elevated level of Bx in *miR-263b^KO^*, we reasoned that overexpression of *Bx* should mimic *miR-263b^KO^*. As expected, overexpression of *Bx* using *Δ263b-Gal4* caused approximately 75% arrhythmicity in DD, which phenocopied the *miR-263b^KO^* (Figure 5E–I and Table 1). In addition, consistent with *miR-263b* overexpression, *Bx* loss-of-function mutation (*Bx^hdpR26^*) exhibited significant reduction of circadian rhythmicity, amplitude of rhythms, and morning anticipation (Figure 5E–I). To determine the temporal requirement of *Bx*, we overexpressed *Bx* during developmental stages or adulthood. Adult-specific overexpression of *Bx* caused ~40% of flies to be arrhythmic, while only a slight and insignificant reduction of rhythms compared to control were observed in overexpression during development (Figure S7, Supplementary Materials). Taken together, these results demonstrate that *Bx* is required for robust circadian locomotor rhythms and is a potential target of *miR-263b*.

### 2.5. *Bx* Regulates the Arborization Rhythms of sLNv Dorsal Projections

**Figure 6:**
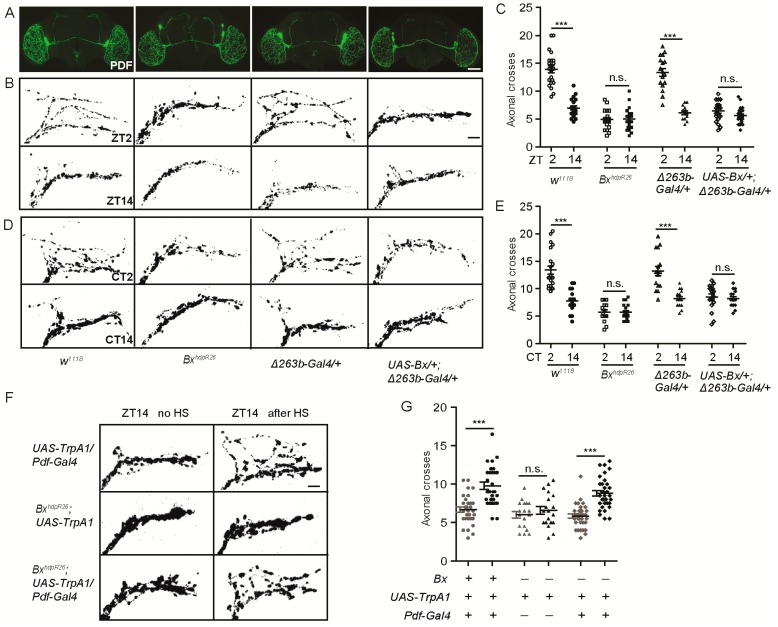
*Bx* regulates the arborization rhythms of sLNv dorsal projections. (**A**) Representative confocal images of brains of the indicated genotypes stained for PDF at ZT2; scale bar, 75 µm. (**B**) Dorsal projections of sLNvs at ZT2 and ZT14. Flies were entrained for at least three days under LD conditions prior to the assay; scale bar, 10 µm. (**C**) Quantification analysis of axonal morphology (fasciculation) of sLNv dorsal termini in LD conditions by Sholl’s analysis. Data represent means ± SEM (*n* = 15–23); n.s. not significant, *** *p* < 0.001 determined by Student’s *t*-test. (**D**) Representative confocal images of the projections at CT2 or CT14 during the second day of DD; scale bar, 10 µm. (**E**) Quantification of axonal morphology of sLNv dorsal termini in DD conditions. Data represent means ± SEM (*n* = 15–21); n.s. not significant, *** *p* < 0.001 determined by Student’s *t*-test. (**F**) Confocal images of sLNv projections from flies stained with anti-PDF at ZT14. Conformation changes in response to 2-h temperature elevation (HS) are shown on the right. The left shows a control not subjected to temperature elevation. (**G**) Quantification of *TrpA1*-induced changes in axonal fasciculation. Data represent means ± SEM (*n* = 19–34); n.s. not significant, *** *p* < 0.001 determined by Student’s *t*-test.

If *miR-263b* regulates circadian rhythms by downregulating *Bx* expression, *Bx^hdpR26^* mutant should phenocopy the circadian structural plasticity defects in *miR-263b* overexpression. We, therefore, examined the dorsal axonal projections of sLNvs in *Bx^hdpR26^* flies. As expected, PDF projections were maintained in the fasciculated state during the day, as well as the night, in *Bx^hdpR26^* flies. At ZT2, *Bx^hdpR26^* showed reduced maximal axonal crosses relative to those in control flies that were not significantly different from those observed at ZT14 (Figure 6A–C). Interestingly, when *Bx* was overexpressed in *miR-263b*-expressing cells, results were similar to those in the *miR-263b^KO^* flies (Figure 6A–C). The rhythmic change in structural plasticity was abolished in *Bx^hdpR26^* or *Bx* overexpression flies in DD (Figure 6D,E). These results support our hypothesis that *miR-263b* regulates the fasciculation–defasciculation state of the PDF termini via effects on *Bx* expression. In addition, activation of PDF cells with *TrpA1* in *Bx^hdpR26^* background resulted in defasciculated sLNv dorsal termini at ZT14 (Figure 6F,G)**.**

### 2.6. *miR-263b* Binding Sites in the *Bx* 3′ UTR Are Essential for Circadian Function of *Bx*

**Figure 7:**
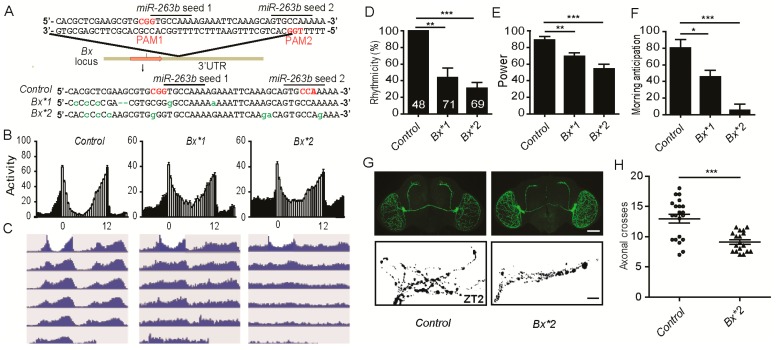
Mutation of the *miR-263b* binding site in the *Bx* 3′ UTR leads to behavioral arrhythmicity. (**A**) Sequence in the *miR-263b* binding site region in the *Bx* 3′ UTR and mutations induced using the CRISPR/Cas9 system. (**B**) Locomotor activity of adult male flies of indicated strains measured during four days of LD cycles and five days of DD. The white and black bars indicate day and night, respectively. (**C**) Actograms showing the average activities on the last day in LD and the five days in DD. Light represents the day, and gray represents darkness. (**D**–**F**) Comparison of (**D**) rhythmicity, (**E**) power, and (**F**) morning anticipation in indicated genotypes. The numbers of tested flies are shown in each column. Data represent means ± SEM (*n* = 48–69). (**G**) Representative confocal images of control and Bx*2 fly brains stained for PDF at ZT2; scale bar, 75 µm for the whole-mount figure, and 10 µm for the magnified images. (**H**) Quantification of axonal morphology from control and Bx*2 flies. Data represent means ± SEM (*n* = 18–21); * *p* < 0.05, ** *p* < 0.01, *** *p* < 0.001 determined by Student’s *t*-test.

To further confirm that *Bx* is a direct *miR-263b* target, we used the CRISPR/Cas9 system to mutate the potential *miR-263b* binding sites within the *Bx* 3′ UTR in flies. It was shown that substitution of one nucleotide in the seed sequence (positions 2–7) is sufficient to reduce miRNA binding to the target mRNA [54]. We recovered two fly lines (Bx*1 and Bx*2) with mutations in the *miR-263b* binding sites (Figure 7A). While Bx*1 had a T-to-G point mutation in the seed sequences of one of the two *miR-263b* binding sites in the *Bx* 3′ UTR, Bx*2 had mutations in both sites. There were also several base changes near the seed sequence that differed in Bx*1 and Bx*2 (Figure 7A). We predicted that, if *miR-263b* suppresses *Bx* abundance through the binding sites in the 3′ UTR, Bx*1 and Bx*2 mutants should phenocopy *miR-263b^KO^*. As expected, both Bx*1 and Bx*2 mutants showed significantly impaired locomotor activity rhythms and reduced power of rhythms (Figure 7B–E). The morning anticipation was also dramatically reduced or abolished, especially in the Bx*2 mutants (Figure 7B–F). Furthermore, similar to the *miR-263b^KO^* flies, Bx*2 mutants also had fasciculated sLNv projections and significantly decreased numbers of axonal crosses at ZT2 (Figure 7G–H). As flies with disrupted *miR-263b* binding sites within *Bx* 3′ UTR show anatomical and behavioral phenotypes similar to those seen in *miR-263b^KO^* flies, we conclude that *miR-263b* regulates circadian rhythm by directly inhibiting *Bx* expression.

## 3. Discussion

As critical post-transcriptional regulators, functions of miRNAs in circadian rhythms are only just being uncovered. Here, we demonstrated that *miR-263b* is critical for circadian locomotor rhythms and axonal structural plasticity of pacemaker neurons. *miR-263b* modulates circadian rhythms via repression of *Bx*. Alterations in levels of *miR-263b* or *Bx* led to behavior arrhythmia and defects of arborization rhythm in sLNvs.

Even though the seed sequences are different, *miR-263a* and *miR-263b* were previously reported to have overlapping function, inhibiting apoptosis in nascent sense organs [44]. In this study, we also tested whether *miR-263a* controls circadian rhythm through regulating *Bx*. Similar to *miR-263b*, *miR-263a* mutants exhibited decrease of circadian rhythmicity (Table 1), indicating a potential circadian function for *miR-263a.* Overexpression of *miR-263a* by its own *Gal4* generated a slightly decreased rhythmicity (Table 1). However, unlike with *miR-263b*, *Bx* was not predicted as a target for *miR-263a*. The in vitro luciferase reporter assay showed no significant repression of *miR-263a* on *Bx* 3′ UTR (Figure S8, Supplementary Materials). It is likely that *miR-263a* uses a different mechanism in the regulation of circadian rhythms.

The mechanisms underlying the structural plasticity of sLNv dorsal projections are poorly understood. This plasticity is controlled by both circadian clock and activity-dependent mechanisms. The arborization rhythm exists even in constant darkness indicating this process is under circadian control. Manipulating the activity of sLNvs can also change the axon plasticity, however. For example, electrically silencing the sLNvs during adulthood can reduce axon complexity [27]. Whether the circadian and activity regulatory mechanisms are independent of each other or interconnected is unknown. Recently, the transcription factor Mef2 was shown to control both circadian- and activity-dependent axonal fasciculation of sLNvs through the neural cell adhesion molecule *Fas2* [28]. This indicates that the two mechanisms might be interconnected through Mef2. Here, we showed that *miR-263b* specifically regulates the circadian structural plasticity by suppressing *Bx* expression. Interestingly, the activity-dependent axonal changes are intact in *miR-263b* and *Bx* mutants. Thus, it is possible that different mechanisms influence circadian regulation and activity-dependent regulation of structural plasticity. However, there is another possibility that *miR-263b* regulates the firing of sLNvs through *Bx*, since our activation of sLNvs by *TrpA1* bypassed this regulation and rescued the neuronal plasticity.

Consistent with a report by Yang et al. [49], our data indicate that there is a cyclic expression of *miR-263b* in fly heads and that this expression is under clock control. Whereas the effect of *miR-263b* on structural plasticity occurred in the early morning, the maximal expression of *miR-263b* was late in the day and in early evening (Figure 1A). This apparent discrepancy between the expression peak and function peak may be due to the mechanism of miRNA action. Most miRNAs function through translational inhibition or degradation of target mRNAs; thus, it is possible that the peak in protein abundance of miRNA targets is opposite the expression peak of the miRNA. In the future, it will be interesting to see whether *Bx* has a peak of abundance at early morning that matches its function.

Where does *miR-263b* function? Evidence suggests that *miR-263b* regulates *Bx* expression in the sLNv cell bodies. That *Bx* is expressed in sLNvs suggests an autonomous mechanism since the *miR-263b* binding sites are critical. *miR-263b* is enriched in central nervous neurons [49]. Our rescue experiments suggest that *miR-263b* is required in the sLNvs for its function in circadian behavior and structural plasticity (Figure 4). However, we cannot exclude non-autonomous contribution of *miR-263b* from PDF-negative neurons or glial cells. It is possible that *miR-263b* non-cell-autonomously regulates *Bx* expression and sLNv activity. A recent study showed that glial inhibition/overexpression of *miR-263b* disrupted circadian behavior rhythms [55]. Unfortunately, overexpression of *miR-263b* using *Repo-Gal4* in *miR-263b* mutants caused lethality problems in our hands; thus, we were not able to directly test the requirement of *miR-263b* with glial specific rescue.

Here, we demonstrated that *miR-263b* regulates *Drosophila* circadian locomotor rhythms and axonal structural plasticity of pacemaker neurons. A similar mechanism may exist in mammals. In the SCN, a group of neurons expressing vasoactive intestinal peptide (VIP) receive retinal light input to synchronize the clock. Interestingly, circadian structural plasticity was found in the glutamatergic synapse on the VIP neurons, which may be important for light entrainment [56]. As a highly conserved miRNA, *miR-263b* has a homologue in vertebrates: *miR-183*. Expression of *miR-183* is enriched in both retina and the pineal gland [57,58]. Furthermore, in the rat pineal gland, *miR-183* is rhythmically expressed with a peak at around ZT12 [57]. This oscillation of *miR-183* suggests a potential circadian function. Bx is a *Drosophila* LIM-only protein. In silico miRNA target prediction with TargetScan identified a highly conserved binding site of *miR-183* in the 3′ UTR of *Lmo3*, a LIM-only protein conserved from rodents to primates [59]. Our results establish *miR-263b* as an important regulator of circadian locomotor behavior and suggest that a highly conserved miRNA–LIM-only protein pathway regulates circadian rhythms in organisms as diverse as flies and mammals.

## 4. Materials and Methods 

### 4.1. Fly Stocks

The following strains were used in this study: *w^1118^*, *Clk^Jrk^*, *Pdf-Gal4*, *UAS-TrpA1*, *tubulin-Gal80^ts^*, *Δ263b-Gal4/TM6B*, *Δ263a-Gal4/CyO*, *Bereft^24^* [50], *UAS-Bx* and *Bx* loss-of-function mutation (*Bx^hdpR26^*) [60]; *UAS-mCD8::GFP*, *Bx-Gal4*, *Repo-Gal4*, *UAS-263b*, and *miR-263b^KO^* (obtained from the Bloomington Stock Center). All flies were raised on standard cornmeal/agar medium at 25 °C under 12-h:12-h LD cycle.

### 4.2. Behavioral Experiments and Analysis

Adult male flies (2–5 days old) were used to test locomotor activity rhythms. Flies were entrained for four days LD cycle at 25 °C, using about 500 lux light intensities, and then released into constant darkness (DD) at 25 °C for at least five days. Locomotor activity was recorded with a TriKinetics Activity Monitor in an I36-LL Percival Incubator (Percival Sciectific, Iowa, USA). FAAS-X software 1.22 was used to analyze behavioral data [61]. Actograms were generated with a signal-processing toolbox for MATLAB. The morning anticipation amplitude was determined by assaying for the locomotor activity as described [43].

### 4.3. Whole-Mount Immunohistochemistry and Quantification

Whole-mount immunohistochemistry of fly brains was done as previously described [16]. For PDF staining, adult flies were entrained to LD for four days and dissected at ZT2 or 14. For PER staining, flies were entrained to LD for four days and then released into DD. Brains were dissected on the second day of DD at six time points. Mouse anti-PDF (1:400, DSHB), rabbit anti-GFP (1:600, ThermoFisher A-6455), rabbit anti-PER (1:1500, gift from Dr. R. Stanewsky), mouse anti-HA (1:400, Sigma H3663), and rabbit anti-HA (1:100, Sigma H6908) antibodies were used. All samples were imaged using a Leica TCS SP8 confocal microscope with a constant laser setting for each time point. In total, 8–10 brains for each genotype were dissected for imaging. ImageJ software [62] was used for GFP, PER, and PDF quantification from at least five brains. For quantification, the average signals of three neighboring background areas were subtracted from the signal intensity in each circadian neuron. Each experiment was conducted three times.

### 4.4. Quantitative Real-Time PCR

Overall, 30–40 flies were collected at the indicated time points, and heads were isolated on dry ice and stored at −80 °C. Total RNA, including miRNA, was purified with miRcute miRNA isolation kits (TIANGEN). Reverse-transcription and real-time PCR of *miR-263b* and *2s rRNA* were performed with first-stand complementary DNA (cDNA) synthesis kits and miRcute miRNA qPCR detection kits (SYBR Green) (TIANGEN). For *miR-263b* the following primer was used: 5′–GCGTTCTCCTTGGCACTGGG. Furthermore, *2s* was used for normalization, and the following primer was used: 5′–TGCTTGGACTACATATGGTTGAGGG. Each experiment was conducted three times.

### 4.5. S2 Cell Luciferase Assay

The full-length 3′ UTR of *Bx* and about 400 base pairs of coding region of *miR-263b* were amplified by PCR using primeSTAR HS DNA Polymerase (TaKaRa). The *Bx* 3′ UTR was cloned into a pAc5.1-firefly luciferase-V5-His vector, and the *miR-263b* coding region was cloned into a pAc5.1-V5-His vector (Invitrogen). The *miR-263b* seed-targeted sequence in the 3′ UTR of *Bx* was mutated from GTGCCAA to CTACTCG using site-directed, ligase-independent mutagenesis [48]. We co-transfected 100 ng of pAc-firefly luc-*Bx* 3′UTR (pAc-firefly luc-*Bx* 3′UTR mutant), 1 µg of pAc-*miR-263b*, 1 µg of pAc-*miR-263a*, and 100 ng of pAc-Renilla luc (transfection control) into S2 cells. Luciferase activity (Dual Luciferase System, Promega, Wisconsin, USA) was measured two days after transfection. Each experiment was conducted three times.

### 4.6. Analysis of Axonal Morphology by Modified Sholl’s Method

The fly genotypes listed in Table 2 were used to analyze activity-dependent changes in axonal morphology. All TrpA1 flies were raised at 18 °C and entrained in LD cycles at 18 °C for at least three days, then shifted to 30 °C at ZT12. Flies were dissected 2 h later (ZT14) and stained with anti-PDF. Structural plasticity was analyzed as reported [28]. Each experiment was conducted three times.

### 4.7. Statistics Analysis

Statistical analysis was performed with SPSS statistics 17.0. The *p*-values were obtained with a Student’s *t*-test and considered not significant (n.s.), or significant at * *p* < 0.05 and extremely significant at *** *p* < 0.001.

## 5. Significance Statement

Circadian rhythms are critical for timing of bodily physiological functions and behavior. MicroRNAs are small non-coding RNAs that play important roles in the post-transcriptional regulation of circadian clocks. Here, we identified the *Drosophila miR-263b* as a new regulator of circadian behavior. Depletion of *miR-263b* abolished morning anticipatory behavior and impaired locomotor rhythmicity. In flies, dorsal axonal projections of the important pacemaker neurons (sLNvs) undergo circadian structural changes, but their physiological importance and the underlying mechanism were unclear. Furthermore, we found *miR-263b* is critical for the structural plasticity of sLNvs. In addition, we identified that *miR-263b* plays its role through inhibition of the LIM-only protein Beadex. Mutating the *miR-263b* binding sites in the Beadex 3′ UTR recapitulated the behavior and anatomical phenotypes of flies lacking *miR-263b.* Our findings reveal that *miR-263b* regulates circadian rhythms and circadian structural plasticity.

## Figures and Tables

**Table 1:** Locomotor activity of flies with altered *miR-263b* and *Bx* levels in constant darkness (DD). SEM—standard error of the mean.

Genotype	*N*	% Rhythmic	Period (h) ± SEM	Power * ± SEM
*w^1118^*	79	98.5 ± 1.5	24.4 ± 0.1	86.0 ± 2.7
*miR-263b^KO^*	60	41.5 ± 10.4	24.3 ± 0.1	58.7 ± 4.8
*Δ263b-Gal4/miR-263b^KO^*	71	39.6 ± 9.0	24.5 ± 0.1	45.7 ± 3.4
*Δ263b-Gal4*, *UAS-263b/miR-263b^KO^*	94	58.0 ± 8.7	24.2 ± 0.1	56.2 ± 3.5
*Δ263b-Gal4/+*	76	96.6 ± 3.3	24.5 ± 0.0	94.1 ± 3.7
*UAS-263b/+*	48	95.8 ± 4.2	23.8 ± 0.1	99.2 ± 3.7
*Δ263b-Gal4/UAS-263b*	45	68.8 ± 2.6	24.2 ± 0.2	61.4 ± 4.5
*UAS-Bx/+*	45	100.0 ± 0.0	24.0 ± 0.1	105.7 ± 3.6
*Bx^hdpR26^*	105	40.1 ± 4.8	25.7 ± 0.2	44.7 ± 2.8
*UAS-Bx/+; Δ263b-Gal4/+*	48	25.1 ± 10.9	24.4 ± 0.4	48.6 ± 6.3
*yw*, *Act5C-Cas9*	48	100.0 ± 0.0	24.4 ± 0.2	89.1 ± 4.0
Bx*1	71	43.6 ± 11.7	24.1 ± 0.1	69.5 ± 4.3
Bx*2	69	31.1 ± 6.7	24.3 ± 0.1	54.3 ± 5.4
*Pdf-Gal4/+*	40	98.0 ± 2.0	24.4 ± 0.1	84.9 ± 3.3
*Pdf-Gal4/+; UAS-263b/+*	60	95.2 ± 4.8	26.1 ± 0.1	86.6 ± 2.5
*UAS-263b*, *miR-263b^KO^/miR-263b^KO^*	42	52.1 ± 4.4	23.9 ± 0.1	57.2 ± 3.5
*Pdf-Gal4/+; miR-263b^KO^/UAS-263b*, *miR-263b^KO^*	57	78.8 ± 5.3	25.5 ± 0.1	67.3 ± 3.0
*tubulin-Gal80^ts^/+; Δ263b-Gal4/+ (29* °C*)*	70	91.8 ± 3.4	24.0 ± 0.1	78.0 ± 3.5
*UAS-263b/+ (29* °C*)*	48	89.9 ± 1.7	23.8 ± 0.1	90.6 ± 4.3
*UAS-Bx/+ (29* °C*)*	31	85.6 ± 8.9	23.9 ± 0.0	88.3 ± 3.5
*tubulin-Gal80^ts^/+; Δ263b-Gal4*, *UAS-263b (29* °C*)*	70	50.0 ± 7.4	24.3 ± 0.1	56.3 ± 4.1
*tubulin-Gal80^ts^/UAS-Bx; Δ263b-Gal4/+ (29* °C*)*	24	41.6 ± 8.3	24.5 ± 0.2	48.6 ± 6.7
*tubulin-Gal80^ts^/+; Δ263b-Gal4/+ (18* °C*)*	62	90.4 ± 6.6	23.6 ± 0.1	103.9 ± 6.5
*UAS-263b/+ (18* °C*)*	68	83.5 ± 4.9	23.7 ± 0.1	72.4 ± 4.9
*UAS-Bx/+ (18* °C*)*	25	85.4 ± 1.4	24.1 ± 0.2	57.4 ± 5.7
*tubulin-Gal80^ts^/+; Δ263b-Gal4*, *UAS-263b (18* °C*)*	50	66.9 ± 3.5	24.5 ± 0.4	55.5 ± 3.9
*tubulin-Gal80^ts^/UAS-Bx; Δ263b-Gal4/+ (18* °C*)*	25	68.3 ± 4.3	23.7 ± 0.2	41.3 ± 4.9
*Bereft^24^*	70	53.2 ± 2.3	23.8 ± 0.1	41.8 ± 2.0
*Δ263a-Gal4/+*	68	96.7 ± 3.3	24.2 ± 0.1	100.8 ± 5.5
*Δ263a-Gal4/UAS-263a*	86	79.7 ± 4.9	23.8 ± 0.1	69.6 ± 4.5

* Power is a measure of rhythm amplitude and corresponds to the height of the periodogram peak above the significance line.

**Table 2:** The fly genotypes for analysis of activity-dependent changes in axonal morphology. ZT—Zeitgeber time; CT—circadian time.

Genotype (Time Point)	*N*	Genotype (Time Point)	*N*
*w^1118^ (ZT2)*	*22*	*w^1118^ (ZT14)*	*20*
*w^1118^ (CT2)*	*21*	*w^1118^ (CT14)*	*15*
*miR-263b^KO^ (ZT2)*	*18*	*miR-263b^KO^ (ZT14)*	*21*
*miR-263b^KO^ (CT2)*	*15*	*miR-263b^KO^ (CT14)*	*18*
*Bx^hdpR26^ (ZT2)*	*21*	*Bx^hdpR26^ (ZT14)*	*20*
*Bx^hdpR26^ (CT2)*	*15*	*Bx^hdpR26^ (CT14)*	*18*
*Δ263b-Gal4/+ (ZT2)*	*17*	*Δ263b-Gal4/+ (ZT14)*	*14*
*Δ263b-Gal4/+ (CT2)*	*16*	*Δ263b-Gal4/+ (CT14)*	*17*
*Δ263b-Gal4/UAS-263b (ZT2)*	*19*	*Δ263b-Gal4/UAS-263b (ZT14)*	*20*
*Δ263b-Gal4/UAS-263b (CT2)*	*16*	*Δ263b-Gal4/UAS-263b (CT14)*	*15*
*UAS-Bx/+; Δ263b-Gal4/+ (ZT2)*	*22*	*UAS-Bx/+; Δ263b-Gal4/+ (ZT14)*	*23*
*UAS-Bx/+; Δ263b-Gal4/+ (CT2)*	*17*	*UAS-Bx/+; Δ263b-Gal4/+ (CT14)*	*17*
*Pdf-Gal4/UAS-mCD8::GFP (ZT2)*	*18*	*Pdf-Gal4/UAS-mCD8::GFP (ZT14)*	*15*
*Pdf-Gal4/UAS-mCD8::GFP;miR-263b^KO^ (ZT2)*	*21*	*Pdf-Gal4/UAS-mCD8::GFP;miR-263b^KO^ (ZT14)*	*17*
*yw*, *Act5C-Cas9 (ZT2)*	*21*	Bx*2 (ZT2)	*18*
*UAS-263b*, *miR-263b^KO^/miR-263b^KO^ (ZT2)*	*17*	*Pdf-Gal4/+; miR-263b^KO^*,*UAS-263b/miR-263b^KO^(ZT2)*	*19*
*Pdf-Gal4/UAS-TrpA1 (ZT14 no HS)*	*31*	*Pdf-Gal4/UAS-TrpA1 (ZT14 after HS)*	*34*
*UAS-TrpA1; miR-263b^KO^ (ZT14 no HS)*	*26*	*UAS-TrpA1; miR-263b^KO^ (ZT14 after HS)*	*31*
*Pdf-Gal4/UAS-TrpA1; miR-263b^KO^ (ZT14 no HS)*	*33*	*Pdf-Gal4/UAS-TrpA1; miR-263b^KO^ (ZT14 after HS)*	*33*
*Bx^hdpR26^; UAS-TrpA1 (ZT14 no HS)*	*18*	*Bx^hdpR26^; UAS-TrpA1 (ZT14 after HS)*	*19*
*Bx^hdpR26^; Pdf-Gal4/UAS-TrpA1 (ZT14 no HS)*	*34*	*Bx^hdpR26^; Pdf-Gal4/UAS-TrpA1 (ZT14 after HS)*	*33*

## Supplementary Materials

The following are available online at https://www.mdpi.com/2073-4409/8/8/923/s1, Figure S1. *miR-263b* expression level in relevant flies. Figure S2. *miR-263b* is required during adulthood, but not development. Figure S3. The molecular pacemaker is not affected in *miR-263b^KO^* flies. Figure S4. PDF levels in the sLNvs soma are not significantly changed under LD conditions. Figure S5. *miR-263b* can drive changes of sLNv axonal projections in short time. Figure S6. Predicted *miR-263b* binding site conservation in the *Bx* 3’ UTR among *Drosophila* species. Figure S7. *Bx* is required during adulthood, but not during development. Figure S8. *miR-263a* cannot inhibit the expression of *Bx* in S2 cells.

## References

[1] Crane B.R., Young M.W. (2014). Interactive features of proteins composing eukaryotic circadian clocks. Annu. Rev. Biochem..

[2] Hardin P.E., Panda S. (2013). Circadian timekeeping and output mechanisms in animals. Curr. Opin. Neurobiol..

[3] Tataroglu O., Emery P. (2015). The molecular ticks of the Drosophila circadian clock. Curr. Opin. Insect Sci..

[4] Dubowy C., Sehgal A. (2017). Circadian Rhythms and Sleep in Drosophila melanogaster. Genetics.

[5] Chiu J.C., Ko H.W., Edery I. (2011). NEMO/NLK Phosphorylates PERIOD to Initiate a Time-Delay Phosphorylation Circuit that Sets Circadian Clock Speed. Cell.

[6] Grima B., Dognon A., Lamouroux A., Chelot E., Rouyer F. (2012). CULLIN-3 Controls TIMELESS Oscillations in the Drosophila Circadian Clock. PLoS Biol..

[7] Kim E.Y., Jeong E.H., Park S., Jeong H.J., Edery I., Cho J.W. (2012). A role for O-GlcNAcylation in setting circadian clock speed. Genes Dev..

[8] Luo W.F., Li Y., Tang C.H.A., Abruzzi K.C., Rodriguez J., Pescatore S., Roshbash M. (2012). CLOCK deubiquitylation by USP8 inhibits CLK/CYC transcription in Drosophila. Genes Dev..

[9] Ko H.W., Jiang J., Edery I. (2002). Role for Slimb in the degradation of Drosophila Period protein phosphorylated by Doubletime. Nature.

[10] Beckwith E.J., Ceriani M.F. (2015). Experimental assessment of the network properties of the Drosophila circadian clock. J Comp. Neurol..

[11] Johard H.A.D., Yoishii T., Dircksen H., Cusumano P., Rouyer F., Helfrich-Forster C., Nassel D.R. (2009). Peptidergic Clock Neurons in Drosophila: Ion Transport Peptide and Short Neuropeptide F in Subsets of Dorsal and Ventral Lateral Neurons. J. Comp. Neurol..

[12] Nitabach M.N., Taghert P.H. (2008). Organization of the Drosophila circadian control circuit. Curr. Biol..

[13] Grima B., Chelot E., Xia R., Rouyer F. (2004). Morning and evening peaks of activity rely on different clock neurons of the Drosophila brain. Nature.

[14] Stoleru D., Peng Y., Agosto J., Rosbash M. (2004). Coupled oscillators control morning and evening locomotor behaviour of Drosophila. Nature.

[15] Guo F., Yu J.W., Jung H.J., Abruzzi K.C., Luo W.F., Griffith L.C., Rosbash M. (2016). Circadian neuron feedback controls the Drosophila sleep-activity profile. Nature.

[16] Zhang Y., Liu Y.X., Bilodeau-Wentworth D., Hardin P.E., Emery P. (2010). Light and Temperature Control the Contribution of Specific DN1 Neurons to Drosophila Circadian Behavior. Curr. Biol..

[17] Yadlapalli S., Jiang C., Bahle A., Reddy P., Eyhofer E.M., Shafer O.T. (2018). Circadian clock neurons constantly monitor environmental temperature to set sleep timing. Nature.

[18] Kaneko H., Head L.M., Ling J.L., Tang X., Liu Y.L., Hardin P.E., Emery P., Hamada F.N. (2012). Circadian Rhythm of Temperature Preference and Its Neural Control in Drosophila. Curr. Biol..

[19] Mohawk J.A., Green C.B., Takahashi J.S. (2012). Central and Peripheral Circadian Clocks in Mammals. Annu. Rev. Neurosci..

[20] Reppert S.M., Weaver D.R. (2002). Coordination of circadian timing in mammals. Nature.

[21] Renn S.C.P., Park J.H., Rosbash M., Hall J.C., Taghert P.H. (1999). A pdf neuropeptide gene mutation and ablation of PDF neurons each cause severe abnormalities of behavioral circadian rhythms in Drosophila. Cell.

[22] Mertens I., Vandingenen A., Johnson E.C., Shafer O.T., Li W., Trigg J.S., Loof A.D., Schoofs L., Taghert P.H. (2005). PDF receptor signaling in Drosophila contributes to both circadian and geotactic behaviors. Neuron.

[23] Helfrichforster C. (1995). The Period Clock Gene Is Expressed in Central-Nervous-System Neurons Which Also Produce a Neuropeptide That Reveals the Projections of Circadian Pacemaker Cells within the Brain of Drosophila-Melanogaster. Proc. Natl. Acad. Sci. USA.

[24] Helfrichforster C., Homberg U. (1993). Pigment-Dispersing Hormone-Immunoreactive Neurons in the Nervous-System of Wild-Type Drosophila-Melanogaster and of Several Mutants with Altered Circadian Rhythmicity. J. Comp. Neurol..

[25] Park J.H., Helfrich-Forster C., Lee G., Liu L., Rosbash M., Hall J.C. (2000). Differential regulation of circadian pacemaker output by separate clock genes in Drosophila. Proc. Natl. Acad. Sci. USA.

[26] Fernandez M.P., Berni J., Ceriani M.F. (2008). Circadian remodeling of neuronal circuits involved in rhythmic behavior. PLoS Biol..

[27] Depetris-Chauvin A., Berni J., Aranovich E.J., Muraro N.I., Beckwith E.J., Ceriani M.F. (2011). Adult-Specific Electrical Silencing of Pacemaker Neurons Uncouples Molecular Clock from Circadian Outputs. Curr. Biol..

[28] Sivachenko A., Li Y., Abruzzi K.C., Rosbash M. (2013). The Transcription Factor Mef2 Links the Drosophila Core Clock to Fas2, Neuronal Morphology, and Circadian Behavior. Neuron.

[29] Depetris-Chauvin A., Fernandez-Gamba A., Gorostiza E.A., Herrero A., Castano E.M., Ceriani M.F. (2014). Mmp1 Processing of the PDF Neuropeptide Regulates Circadian Structural Plasticity of Pacemaker Neurons. PLoS Genet..

[30] Petsakou A., Sapsis T.P., Blau J. (2015). Circadian Rhythms in Rho1 Activity Regulate Neuronal Plasticity and Network Hierarchy. Cell.

[31] Gunawardhana K.L., Hardin P.E. (2017). VRILLE Controls PDF Neuropeptide Accumulation and Arborization Rhythms in Small Ventrolateral Neurons to Drive Rhythmic Behavior in Drosophila. Curr. Biol..

[32] Bartel D.P. (2004). MicroRNAs: Genomics, biogenesis, mechanism, and function. Cell.

[33] Colpaert R.M.W., Calore M. (2019). MicroRNAs in Cardiac Diseases. Cells.

[34] Falzone L., Lupo G., Rosa G.R.M.L., Crimi S., Anfuso C.D., Salemi R., Candido S., Libra M., Candido S. (2019). Identification of Novel MicroRNAs and Their Diagnostic and Prognostic Significance in Oral Cancer. Cancers.

[35] Tomankova T., Petrek M., Kriegova E. (2010). Involvement of microRNAs in physiological and pathological processes in the lung. Respir. Res..

[36] Mendoza-Viveros L., Chiang C.K., Ong J.L.K., Hegazi S., Cheng A.H., Bouchard-Cannon P., Fana M., Lowden C., Zhang P., Bothorel B. (2017). miR-132/212 Modulates Seasonal Adaptation and Dendritic Morphology of the Central Circadian Clock. Cell Rep..

[37] Xue Y.B., Zhang Y. (2018). Emerging roles for microRNA in the regulation of Drosophila circadian clock. BMC Neurosci..

[38] Cheng H.Y.M., Papp J.W., Varlamova O., Dziema H., Russell B., Curfman J.P., Nakazawa T., Shimizu K., Okamura H., Impey S. (2007). microRNA modulation of circadian-clock period and entrainment. Neuron.

[39] Yoo S.H., Kojima S., Shimomura K., Koike N., Buhr E.D., Furukawa T., Ko C.H., Gloston G., Ayoub C., Nohara K. (2017). Period2 3 ‘-UTR and microRNA-24 regulate circadian rhythms by repressing PERIOD2 protein accumulation. Proc. Natl. Acad. Sci. USA.

[40] Chen W.F., Liu Z.X., Li T.J., Zhang R.F., Xue Y.B., Zhong Y., Bai W.W., Zhou D.S., Zhao Z.W. (2014). Regulation of Drosophila circadian rhythms by miRNA let-7 is mediated by a regulatory cycle. Nat. Commun..

[41] Chen X., Rosbash M. (2016). mir-276a strengthens Drosophila circadian rhythms by regulating timeless expression. Proc. Natl. Acad. Sci. USA.

[42] Kadener S., Menet J.S., Sugino K., Horwich M.D., Weissbein U., Nawathean P., Vagin V.V., Zamore P.D., Nelson S.B., Rosbash M. (2009). A role for microRNAs in the Drosophila circadian clock. Genes Dev..

[43] Zhang Y., Emery P. (2013). GW182 Controls Drosophila Circadian Behavior and PDF-Receptor Signaling. Neuron.

[44] Luo W.Y., Sehgal A. (2012). Regulation of Circadian Behavioral Output via a MicroRNA-JAK/STAT Circuit. Cell.

[45] Vodala S., Pescatore S., Rodriguez J., Buescher M., Chen Y.W., Weng R.F., Cohen S.M., Rosbash M. (2012). The Oscillating miRNA 959-964 Cluster Impacts Drosophila Feeding Time and Other Circadian Outputs. Cell Metab..

[46] Garaulet D.L., Sun K.L., Li W.H., Wen J.Y., Panzarino A.M., O’Neil J.L., Hiesinger P.R., Young M.W., Lai E.C. (2016). miR-124 Regulates Diverse Aspects of Rhythmic Behavior in Drosophila. J. Neurosci..

[47] Zhang Y., Lamba P., Guo P.Y., Emery P. (2016). miR-124 Regulates the Phase of Drosophila Circadian Locomotor Behavior. J. Neurosci..

[48] Niu Y., Liu Z., Nian X., Xu X., Zhang Y. (2019). miR-210 controls the evening phase of circadian locomotor rhythms through repression of Fasciclin 2. PLoS Genet..

[49] Yang M., Lee J.E., Padgett R.W., Edery I. (2008). Circadian regulation of a limited set of conserved microRNAs in Drosophila. BMC Genomics.

[50] Hilgers V., Bushati N., Cohen S.M. (2010). Drosophila microRNAs 263a/b Confer Robustness during Development by Protecting Nascent Sense Organs from Apoptosis. PLoS Biol..

[51] Gorostiza E.A., Depetris-Chauvin A., Frenkel L., Pirez N., Ceriani M.F. (2014). Circadian Pacemaker Neurons Change Synaptic Contacts across the Day. Curr. Biol..

[52] Hamada F.N., Rosenzweig M., Kang K., Pulver S.R., Ghezzi A., Jegla T.J., Garrity P.A. (2008). An internal thermal sensor controlling temperature preference in Drosophila. Nature.

[53] Tsai L.T.Y., Bainton R.J., Blau J., Heberlein U. (2004). Lmo mutants reveal a novel role for circadian pacemaker neurons in cocaine-induced behaviors. PLoS Biol..

[54] Brennecke J., Stark A., Russell R.B., Cohen S.M. (2005). Principles of microRNA-target recognition. PLoS Biol..

[55] You S., Fulga T.A., Van Vactor D., Jackson F.R. (2018). Regulation of Circadian Behavior by Astroglial MicroRNAs in Drosophila. Genetics.

[56] Girardet C., Blanchard M.P., Ferracci G., Leveque C., Moreno M., Francois-Bellan A.M., Becquet D., Bosler O. (2010). Daily changes in synaptic innervation of VIP neurons in the rat suprachiasmatic nucleus: Contribution of glutamatergic afferents. Eur. J. Neurosci..

[57] Clokie S.J.H., Lau P., Kim H.H., Coon S.L., Klein D.C. (2012). MicroRNAs in the Pineal Gland miR-483 regulates melatonin synthesis by targeting arylalkylamine N-acetyltransferase. J. Biol. Chem..

[58] Xu S.B., Witmer P.D., Lumayag S., Kovacs B., Valle D. (2007). MicroRNA (miRNA) transcriptome of mouse retina and identification of a sensory organ-specific miRNA cluster. J. Biol. Chem..

[59] Dambal S., Shah M., Mihelich B., Nonn L. (2015). The microRNA-183 cluster: The family that plays together stays together. Nucleic Acids Res..

[60] Bejarano F., Smibert P., Lai E.C. (2010). miR-9a prevents apoptosis during wing development by repressing Drosophila LIM-only. Dev. Biol..

[61] Grima B., Lamouroux A., Chelot E., Papin C., Limbourg-Bouchon B., Rouyer F. (2002). The F-box protein Slimb controls the levels of clock proteins Period and Timeless. Nature.

[62] Schneider C.A., Rasband W.S., Eliceiri K.W. (2012). NIH Image to ImageJ: 25 years of image analysis. Nat. Methods.

