# Supplementary material for: miR-263b Controls Circadian Behavior and the Structural Plasticity of Pacemaker Neurons by Regulating the LIM-Only Protein Beadex
Source: Cells. 2019 Aug 18;8(8):923. doi: 10.3390/cells8080923 (PMC6721658; doi:10.3390/cells8080923)
Supplement: Supplementary file 1 [file cells-08-00923-s001.pdf]

# miR-263b Controls Circadian Behavior and the Structural Plasticity of Pacemaker Neurons by Regulating the LIM-Only Protein Beadex

**Xiaoge Nian[1,2], Wenfeng Chen[2,3], Weiwei Bai[1], Zhangwu Zhao[1,]* and Yong Zhang[2,]***

[1] Department of Entomology and MOA Key Lab of Pest Monitoring and Green Management, College of Plant Protection, China Agricultural University, Beijing, 100193, China
[2] Department of Biology, University of Nevada Reno, Reno, NV 89557, USA
[3] Institute of Life Sciences, Fuzhou University, Fuzhou 350108, China
* Correspondence: zhaozw@cau.edu.cn (Z.Z.); yongzhang@unr.edu (Y.Z.)

**Supplementary Materials**

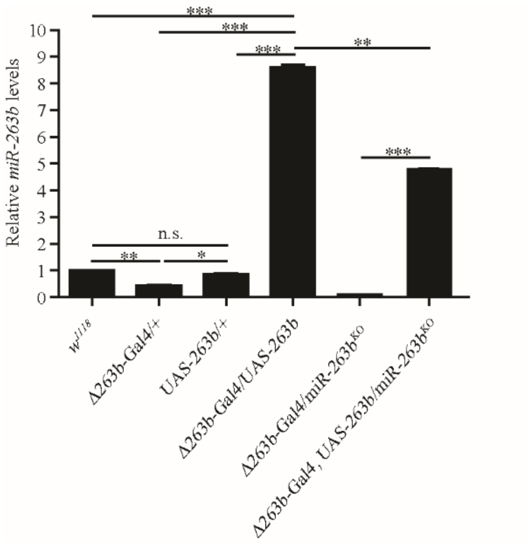


**Figure S1.** *miR-263b* expression level in relevant flies. Quantitative real-time PCR analysis of total RNA prepared from adult brains at ZT13. The relative expression levels were normalized to *2s* RNA levels and were further normalized to $w^{1118}$ control. Data represent means ± SEM; n.s. not significant, * $p < 0.05$, ** $p < 0.01$, *** $p < 0.001$ determined by Student's *t*-test.

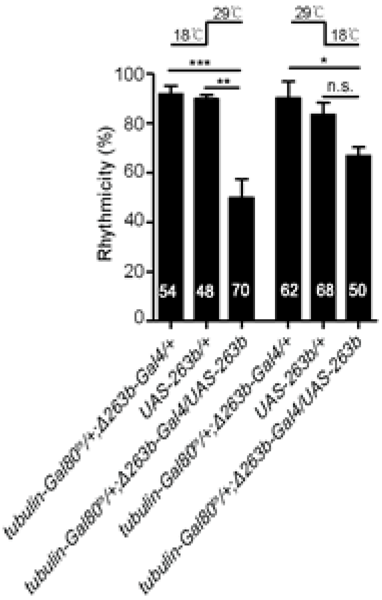


**Figure S2.** *miR-263b* is required during adulthood, but not development. Left, flies were grown at 18 °C to inhibit *miR-263b* expression during development and transferred at 29 °C after eclosion to allow *miR-263b* overexpression in adult flies. Right, flies were grown at 29 °C to inactivate *Gal80*, thus allowing *miR-263b* overexpression during development, and then transferred to 18 °C to block *miR-263b* overexpression after eclosion. Data represent means ± SEM; n.s. not significant, * $p < 0.05$, ** $p < 0.01$, *** $p < 0.001$ determined by Student's *t*-test. Percentage of rhythmicity is indicated above the bars, and the number of flies tested is shown in the bars.

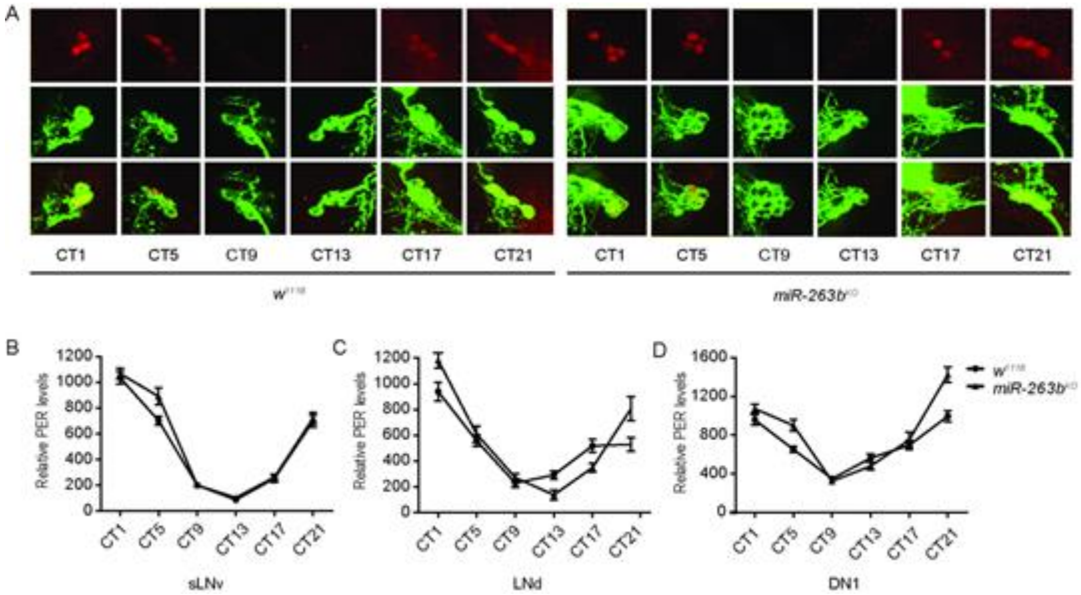


**Figure S3.** The molecular pacemaker is not affected in *miR-263b*$^{KO}$ flies. The molecular pacemaker is not affected in *miR-263b*$^{KO}$ flies. (A) Representative confocal images of sLNvs from $w^{1118}$ and *miR-263b*$^{KO}$ flies dissected at six time points (circadian time, CT) during the second day of DD and stained with anti-PDF (green) and anti-PER (red) antibodies; scale bar, 10 μm. (B–D) Quantification of PER staining in sLNvs, LNds, and DN1s at different circadian time points. Data represent means ± SEM ($n$ = 16–19).

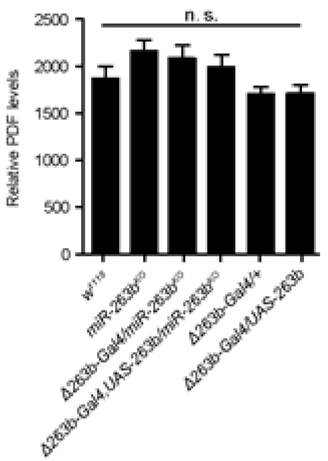


**Figure S4.** PDF levels in the sLNvs soma are not significantly changed under LD conditions. Quantification of PDF staining in sLNvs at ZT2. Data represent means ± SEM ($n$ = 18–21); n.s. not significant determined by Student's $t$-test.

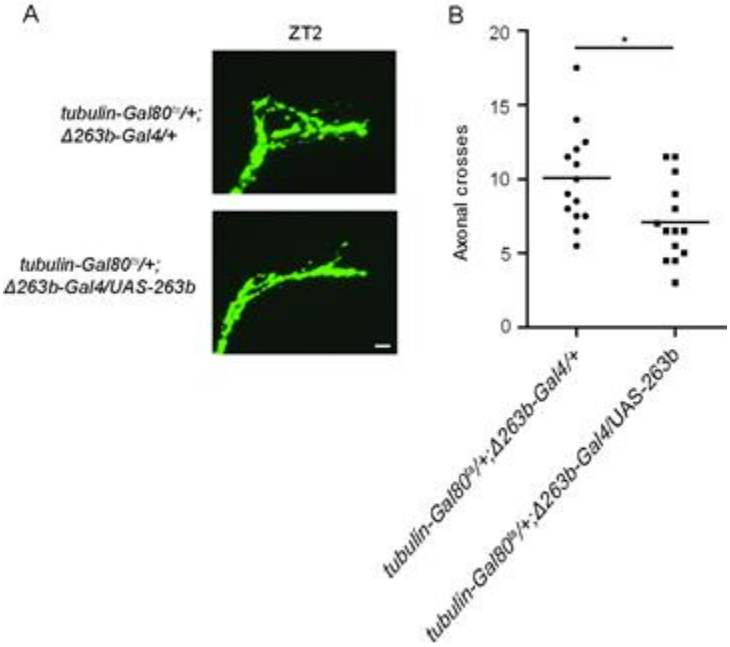


**Figure S5.** *miR-263b* can drive changes of sLNv axonal projections in short time. (A) Representative images of sLNv dorsal projections from the indicated genotypes stained with anti-PDF at ZT2; scale bar, 10 μm. (B) Quantification of axonal morphology (fasciculation) of sLNv dorsal termini in LD conditions by Sholl's analysis. Data represent means ± SEM ($n$ = 14); * $p < 0.05$ determined by Student's $t$-test.

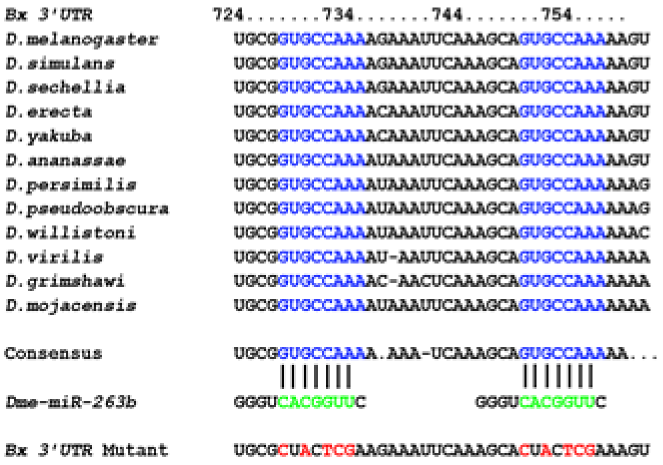


**Figure S6.** Predicted *miR-263b* binding site conservation in the *Bx* 3′ UTR among *Drosophila* species. Blue letters indicate conserved sequences, green letters indicate *miR-263b* seed region, and red letters indicate positions of mutations in *Bx* 3′ UTR made for S2 cell reporter gene assay.

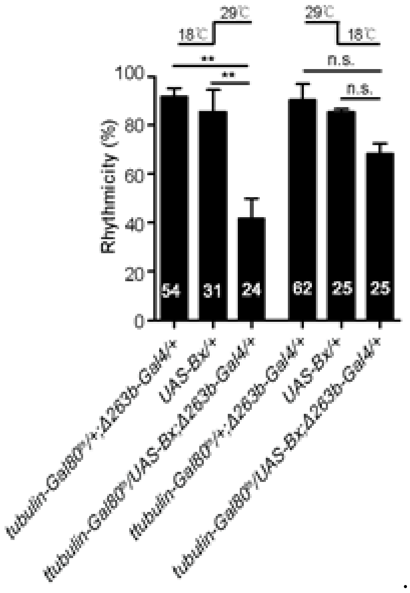


**Figure S7.** *Bx* is required during adulthood, but not during development. Left, flies were grown at 18 °C to inhibit *Bx* expression during development and transferred at 29 °C after eclosion to allow *Bx* overexpression in adult flies. Right, flies were grown at 29 °C to inactivate $Gal80^{ts}$, thus allowing *Bx* overexpression during development, and then transferred to 18 °C to block *Bx* overexpression after eclosion. Data represent means ± SEM; n.s. not significant, ** $p < 0.01$, determined by Student's *t*-test. Percentage of rhythmicity is indicated above the bars, and the number of flies tested is shown in the bars.

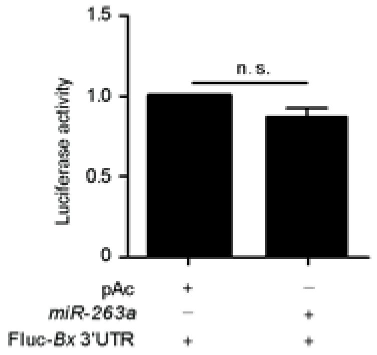


**Figure S8.** *miR-263a* cannot inhibit the expression of *Bx* in S2 cells. pAC or pAC-*miR-263a* was co-transfected with pAc-fluc-*Bx* 3′ UTR into S2 cells. After two days, luciferase activity was quantified. For each condition, a normalized firefly/*Renilla* luciferase value is plotted with SEM.
